# A New Look at Shelter 131/51 in the Natufian Site of Eynan (Ain-Mallaha), Israel

**DOI:** 10.1371/journal.pone.0130121

**Published:** 2015-07-08

**Authors:** Gil Haklay, Avi Gopher

**Affiliations:** Sonia and Marco Nadler Institute of Archaeology, Tel Aviv University, Tel Aviv, Israel; University College Dublin, IRELAND

## Abstract

In the past 25 years since the reconstruction of Shelter 131 of Eynan was suggested by Francois Valla, its image has become almost iconic—a highly cited symbol of early sedentism constituting a significant part of our knowledge on early stone constructions and the people behind them. A new look at the architectural remains and the stratigraphy resulted in an alternative reconstruction, essentially different than the one we have come to know. We used spatial (architectural-geometrical) analysis in order to study the relationships between the different architectural elements and to test our hypothesis that the series of postholes may have not pertained to the upper floor 131 of Layer IV as suggested by Perrot and Valla, but rather to the successive occupational and architectural episode. The association of the postholes with Wall 51 of Layer III sheds new light on the architectural remains revealing their geometric design, an important characteristic of Early Natufian Architecture, the meaning and implications of which we shortly discuss.

## Introduction

The Natufian culture was first recognized by Dorothy Garrod following her 1928 excavations at Shukba cave and later at El-Wad cave and terrace. More Natufian sites were discovered later, but it was not until the excavations directed by Jean Perrot at Eynan (Ain-Mallaha) in the late 1950's and the 1960's, that substantial architectural features were uncovered and the innovation of stone construction became part and parcel of the Natufian repertoire. Prior to the Natufian, stone architecture which is generally associated with sedentism was rare [1, 2] and it later became a hallmark of the Neolithic period.

### The site of Eynan

The site of Eynan is located about 200m south to the spring of Eynan, the largest spring of the Hula Valley in the northern Jordan rift. It is situated at the foot of the Naftali Mountains (the upper Galilee eastern slope), on a hillside that locally slopes to the north facing the spring. Natufian occupations with associated architectural remains and burial grounds were dated to all three Natufian cultural phases [Natufian cultural sub-division: Early, Late, and Final Natufian (after F. Valla)] spanning some 3,000 years (from ca. 15,000 to 12,000cal years BP). The settlement was estimated to cover about 2,000 sq m [3] and to inhabit a population of 50–100 people. Semi-subterranean curvilinear structures [built architectural features] made of undressed limestone characterized the site throughout its history. Their construction usually consisted of cutting into the slope and building retaining walls in order to support the surrounding sloping ground. The superstructure (roof) of these shelters [a combination of associated structures and floors] was presumed to have been made of organic material.

It is of note that in the latest series of excavations at Eynan by Valla and Khalaily in the late 1990's and in the 2000's devoted mainly to the later phases of the stratigraphy of the site, exposed a series of curvilinear, relatively small stone structures of a somewhat different nature than the early Natufian ones [4–9].

### The Early Natufian architectural remains

**Fig 1:**
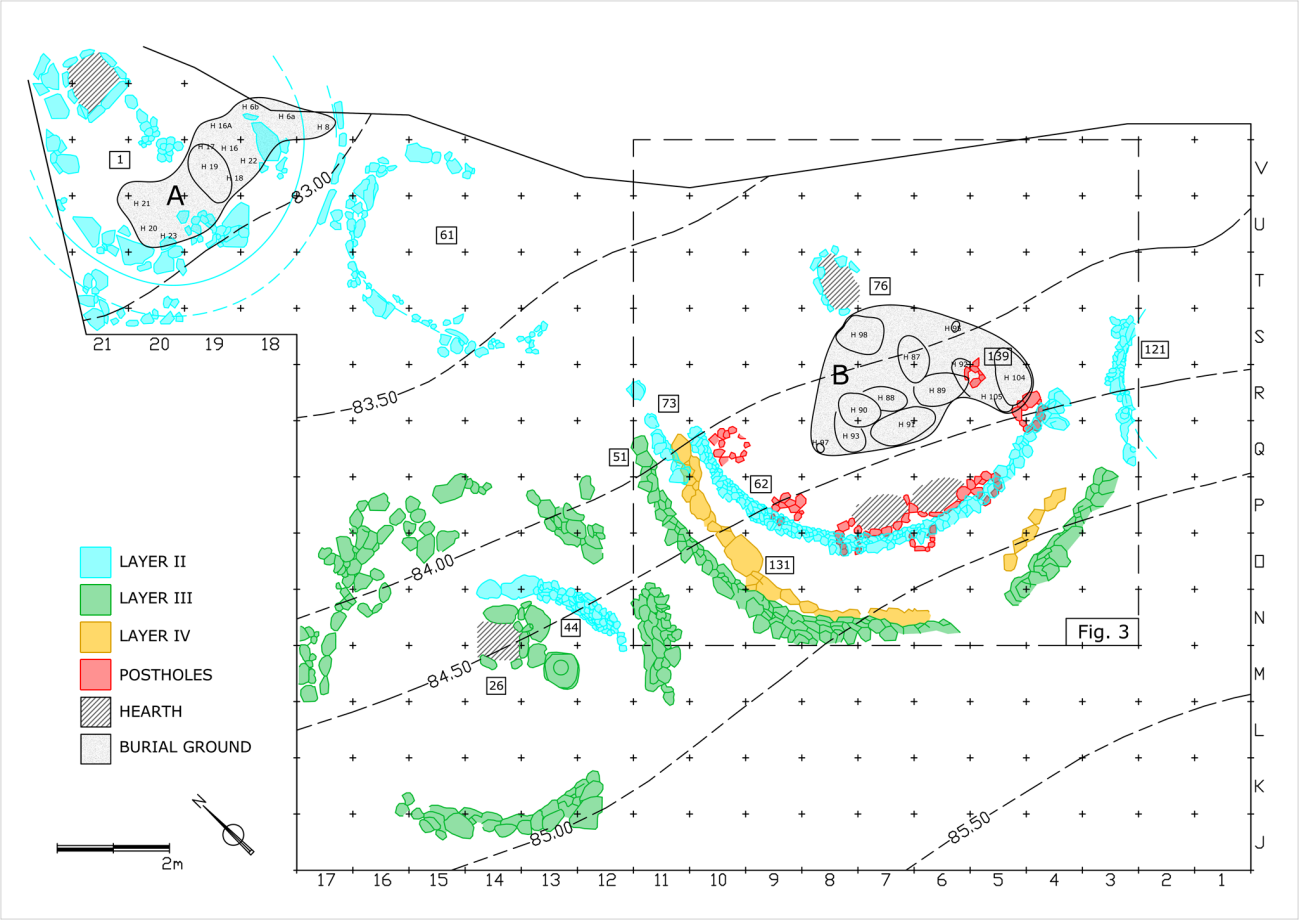
Plan of the Early Natufian architectural remains. Modified from [3, 10, 11, 17, 18].

A total of ca. 120 sq m were excavated down to the lower Early Natufian Layers [stratigraphic sub-divisions, Layers I-IV (after J. Perrot)] III and IV, revealing the two superimposed Shelters 131(Layer IV) and 51(Layer III), Shelter 26 (Layer III) to their west, and two burial grounds each containing10 and 12 burials within an area of approximately 5–7 sq m each. "Cemetery B" (after Perrot [10]), presumably the older one, is located in the area of Shelter 131/51, while "Cemetery A" is located in the area of Shelter 1 of Layer II that represents the last of the Early Natufian occupations (Fig 1A and 1B). Layer II also contains structures 121, 61, 44 and shelter 62—a third concentric wall in the area of Shelters 131/51. All large Shelters of the Early Natufian Layers were first interpreted as dwellings [11] even though they differ in characteristics and size. Shelter 1 has no perimeter wall but a lime plastered bench (5 m in diameter), while Shelter 131/51 is presumed to have been a semi-circular structure (8–9 m in diameter) and features a series of postholes. It has been suggested that Shelter 1 had a ritualistic shamanic purpose (e.g., [12]) or had been an actual funerary monument [13], and that the multiple hearths of Shelter 131 seem to have been used for communal activities (e.g., [13]).

### Shelter 131/51

**Fig 2:**
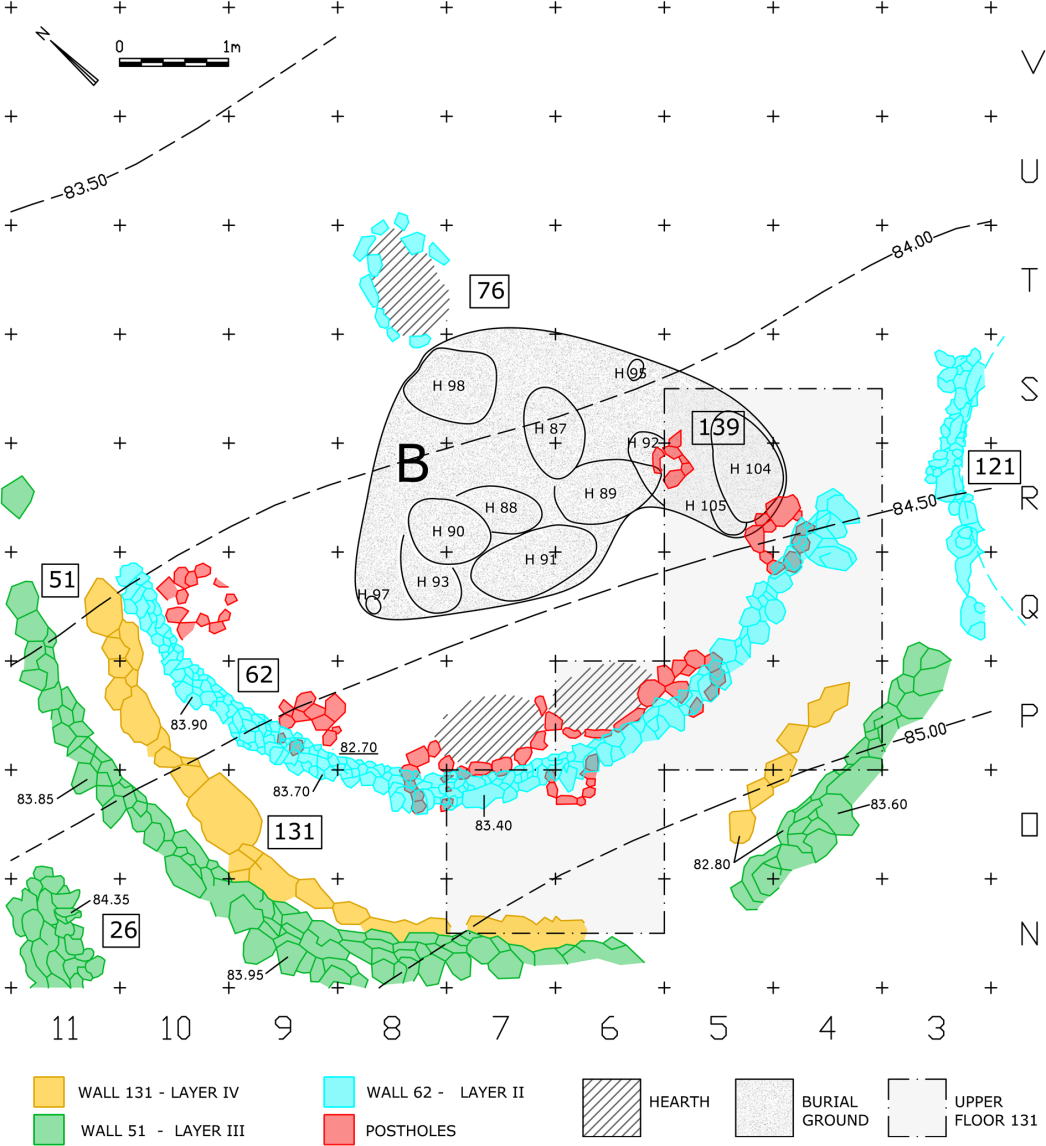
Plan of the Early Natufian architectural remains in the area of Shelter 131/51. Modified from [3, 10, 11, 17, 18].

**Fig 3:**
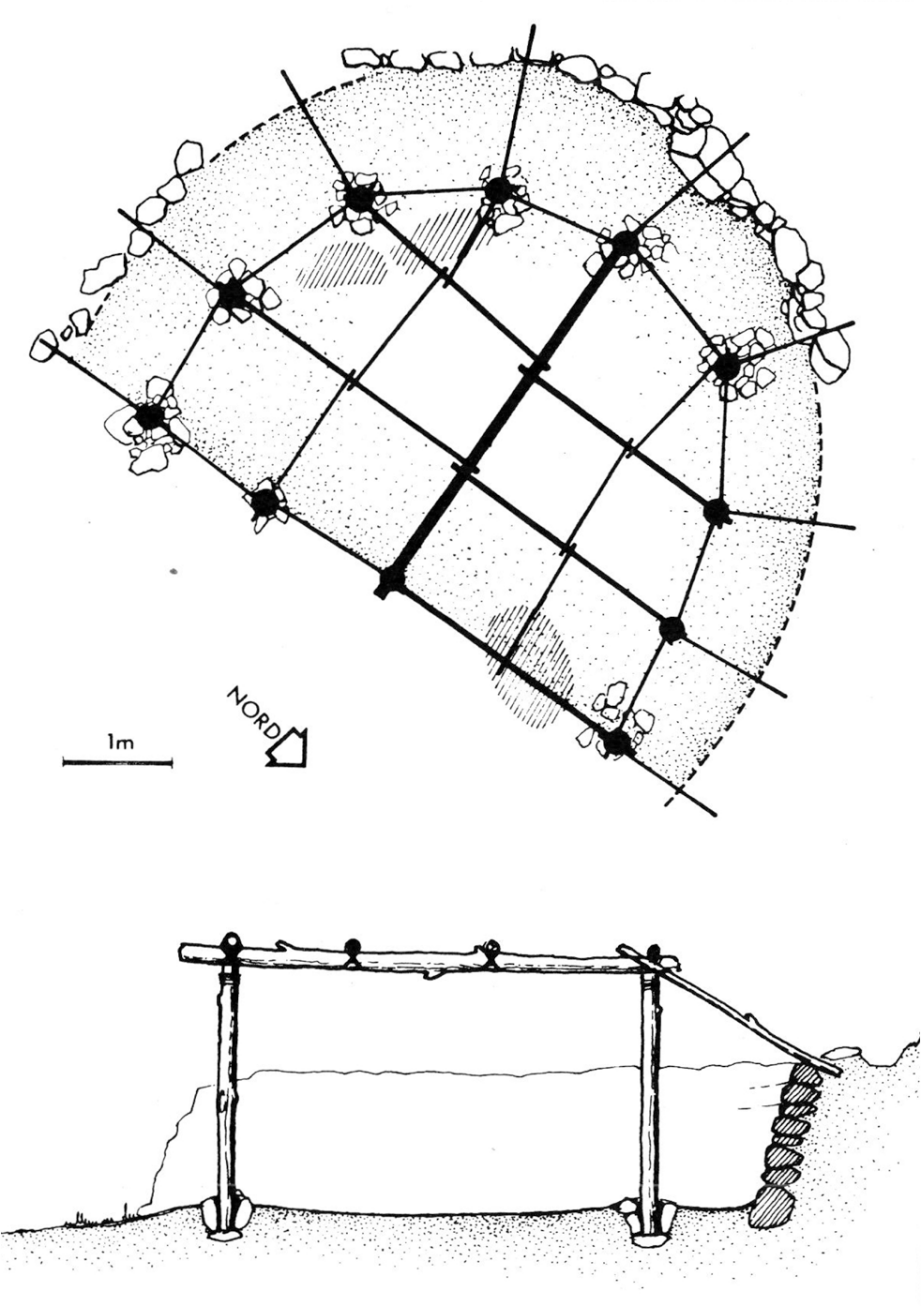
Reconstruction of Shelter 131. After Valla [14] (note the three reconstructed postholes).

Shelter 131/51 is our main interest here (Fig 2, enlarged from Fig 1). According to Valla, the architectural remains and stratigraphy in this part of the site suggests a sequence of construction episodes [architectural and occupational events], occupations and abandonments [14] (Fig 3). The floors were usually not paved but every structure is associated with at least one living floor up to 10-15cm thick. These living floors were traced by the excavators following changes of sediment and the dispersed ashes from the associated hearths. Valla suggested that Shelter 62 of Layer II, the latest of the Early Natufian shelters in this area, was built at a time when all traces of former constructions were covered up, and the fact that it is generally concentric to the outline of 131/51 can be explained by the remaining ground depression that made that location favorable. Wall 51 and its associated floor of Layer III represent, according to Valla, an episode of rebuilding after abandonment and collapse of the earlier Wall 131 of Layer IV, this time cutting deeper into the slope. Layer IV in this part of the site contains the remains of Wall 131 and two living floors (separated by 10cm of sediment), out of which the upper floor (mainly the eastern parts of it) was studied extensively by Valla [14]. The Upper Floor 131 is not only rich in artifacts, but also contains a pair of adjoining constructed hearths delineated by stone arches open towards the north, and the upper stone crowns of seven stone-packed postholes extending deeper into the soil. The packing of stones ensures that the post remains vertical when horizontal loads (generated by an inclined roof beam, winds, etc) are applied to it, by increasing the density of the ground along its underground part. Six of the postholes seem to outline an arch. The assignment of the postholes to Floor 131 was based on the elevation of their uppermost stones, but considering the many construction activities that took place at this location (which always included digging), such an assumption should not be necessarily adopted. In fact, Valla points out that one of the postholes (142 O / 8) was clearly outcropping above the surface considered relevant [14]. Even though the elevations of each posthole and the elevations along the base of each wall were not fully published, it is sufficient to say that under these circumstances their stratigraphic assignment cannot be solely based on their elevation. Other aspects of the remains such as their spatial arrangement should be examined too in order to successfully assign the postholes to the corresponding shelter. Perrot [15] (and more recently, Samuelian [16]) described the postholes as installed 1m in front of, and parallel to Wall 131. This description is not fully accurate. We have noted that the distances from the center of the postholes to Wall 131 vary between 0.6 to 1.25 m, while the distances of these postholes to wall 51 seem to be constant (S1 Fig). This actually triggered our inquiry of the matter.

In an attempt to disclose the relationships between the different architectural elements in the area of Shelter 131/51, we applied a spatial (architectural-geometrical) analysis using a statistical (standard distance deviation) method. In this method, a center point is identified relative to each curvilinear wall outline (structure). The resulting center points provide significant insights and an alternative suggested interpretation regarding the sequence of events at this location. Not without hesitations, and based on our conviction that the geometric pattern is compelling, we present our interpretation below.

## Spatial Analysis: Methods and Results

**Fig 4:**
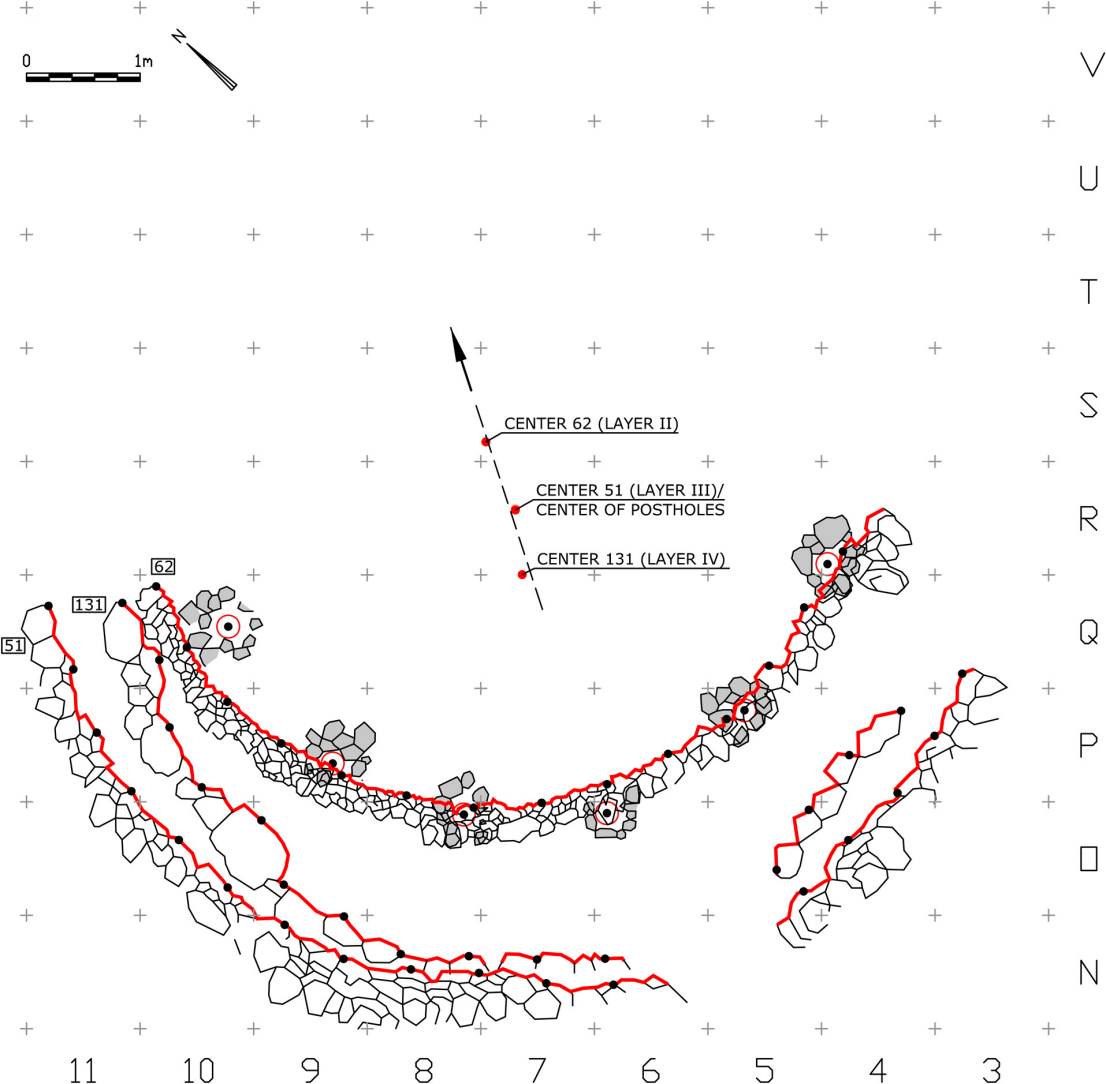
Architectural-geometrical analysis and results.

**Fig 5:**
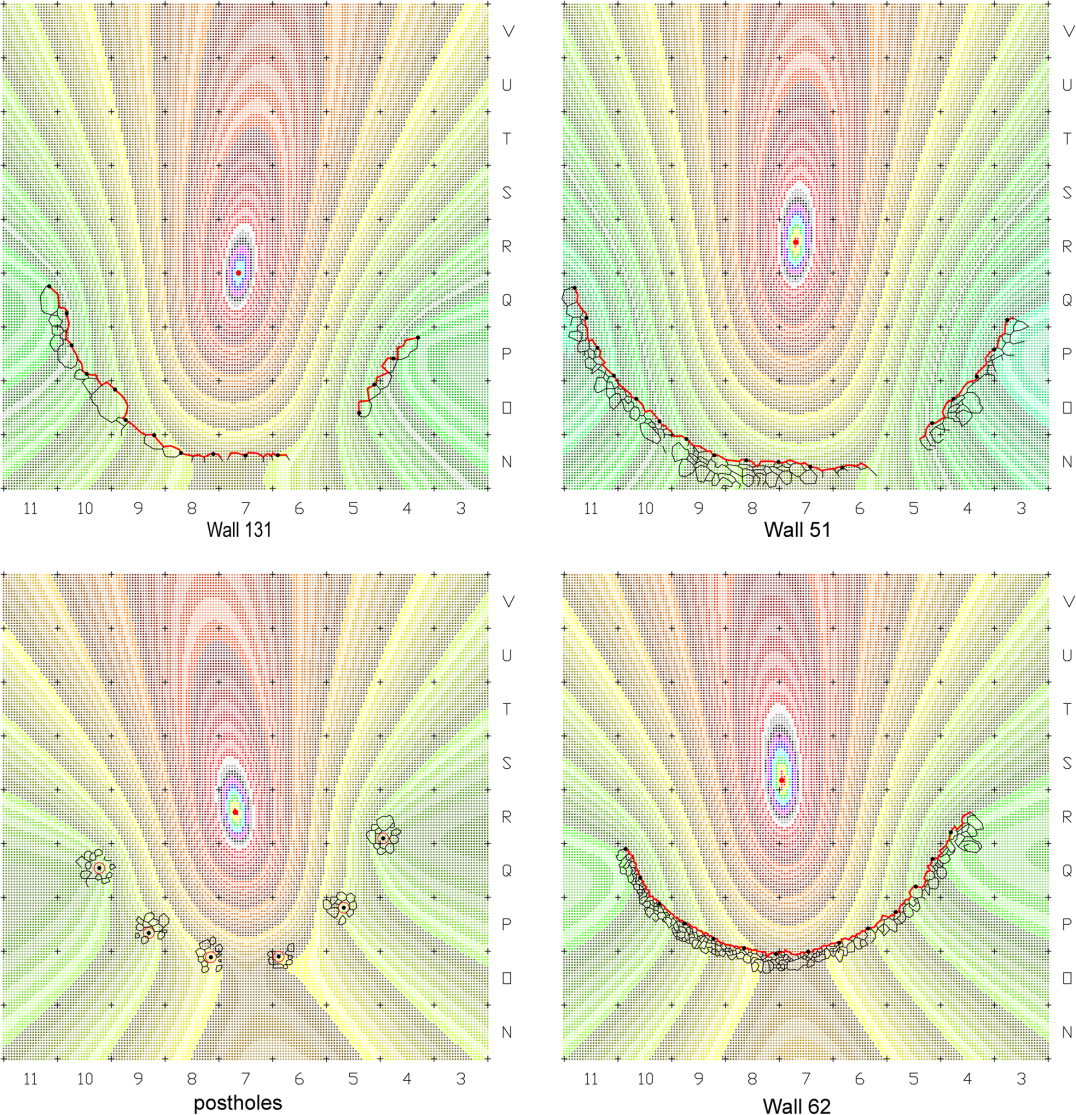
Visualization of the centers' calculation.

The analysis was made on drawings after Jean Perrot [3, 10, 11, 17, 18]. Each curved wall was represented by a set of points, evenly spaced at an interval of 60cm along the line drawing that marks the wall's inner bottom edge (Fig 4). An algorithm that searches for a statistical center point in which the standard distance deviation to the given points is minimal (S2 Fig) was applied separately to each structure and to the arch of six postholes marked by their centers (Fig 5). This statistical method compensates for the roughness of the undressed stones and for minute survey and drawing inaccuracies. The resulted center points can be considered a property of the structures and were most probably spatially perceived by the builders and users of each structure. The analysis of the statistical centers yielded a common point (in a precision of 1cm) as the approximate center of Wall 51 and of the series of postholes, while the other two centers of Wall 131 and Wall 62 were located 60cm to the south and 65cm to the north of that location (Fig 4).

We emphasize the fact that the results of the spatial analysis do not simply indicate that the postholes better match Wall 51 than Wall 131, but rather that the concentricity of Wall 51 and the arch of postholes is precise. Furthermore, considering the sequence of construction [1). digging into the slope, 2). building a wall, 3). installing the postholes], it is a fact that the postholes were built with reference (conscious or not) to one of the walls, which again makes it plausible that the observed match between the features that turned out to be exact, is meaningful.

**Fig 6:**
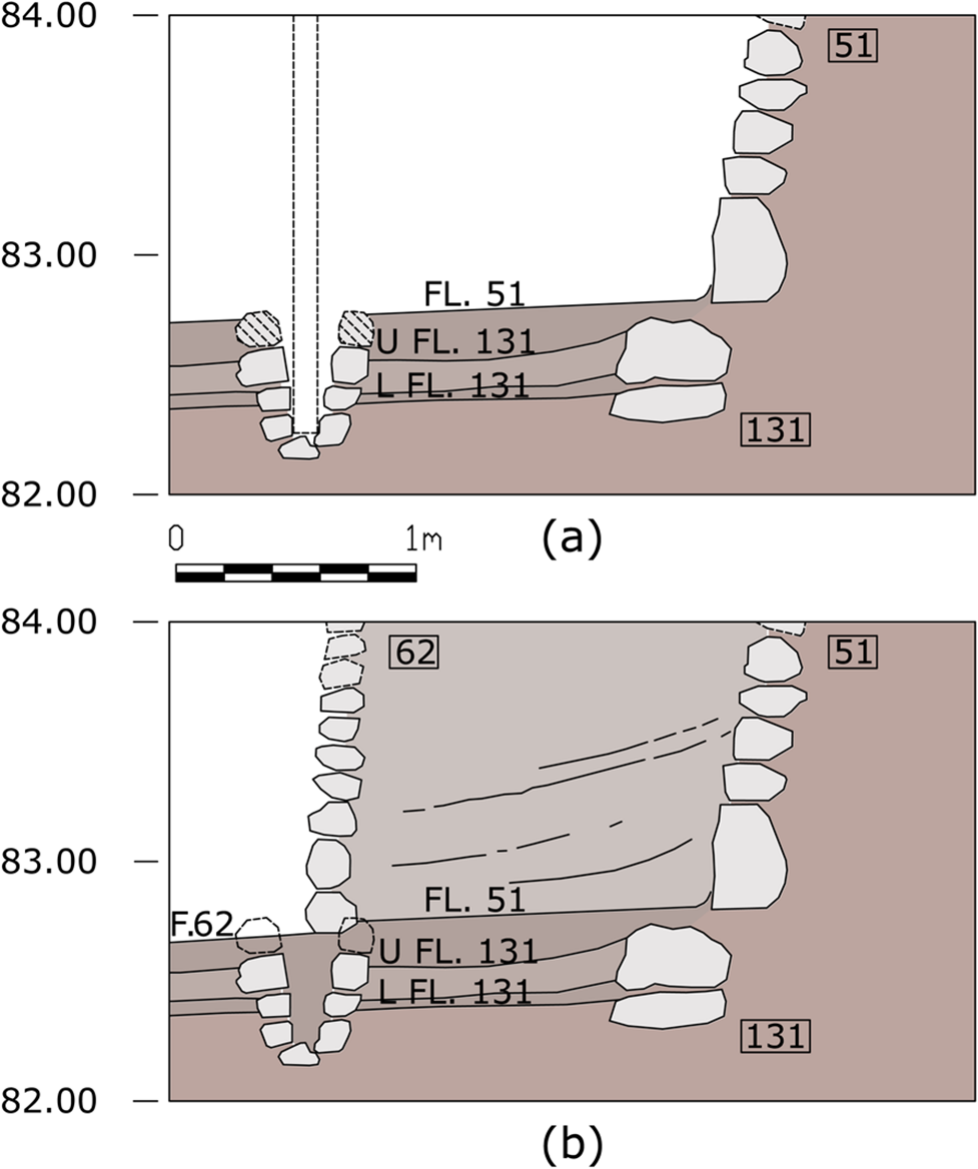
Reconstruction of a posthole. (a) Section showing the reconstruction of a posthole built from floor 51 (note different shading of the stones). (b) Section showing the partial destruction of the posthole due to the construction of Shelter 62.

The difference in elevation between the top of the postholes stones and the bottom of Wall 51 (some 15cm [15]), can be explained by the successive construction of Shelter 62 whose floor, according to Valla, penetrated and destroyed the floor related to Wall 51 [14]. Wall 62 was built right on top of four of the postholes and right behind the other two. It is not unreasonable to assume that during the preparatory earthworks for the construction of Wall 62, the top crown stones of the postholes were shaved off, leaving holes in the ground that were filled with sediment in order to level the surface (Fig 6). This may be the case of the curved double hearth installation as well (Fig 2). These hearths are strongly linked to the layout of the postholes and they must have been dug and built at the same architectural episode, maybe on the spot of an earlier hearth of Shelter 131.

**Fig 7:**
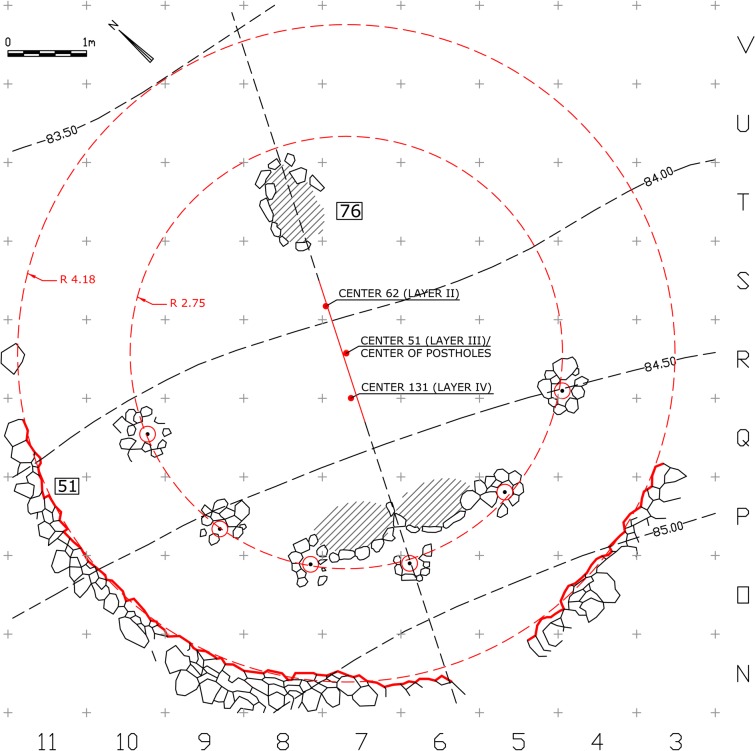
Architectural-geometrical analysis and results.

The association of the postholes with Wall 51 ultimately revealed a simple geometric pattern that enabled us to reconstruct the suggested plan. The regression line calculated from the resulting set of center points indicates the axis of construction at this location along the Early Natufian occupations (Fig 4). This axis which seems to align with the direction of the slope (possibly in ancient times as well), might indicate a possible facing direction. Considering the Early Natufian construction techniques, the direction of the slope should ideally be opposite to the direction of interest, which in Eynan’s case might have been the spring to the North. This North-South axis was ultimately embedded in the spatial organization of Shelter 51 (Fig 7) connecting a series of features including the southern posthole, the symmetry axis of the double hearth installation (described by Valla as the true center of the building [14], the common center of Wall 51 and of the concentric arch of postholes, and hearth 76 that was initially attributed to Shelter 51 [11] and later suggested to have existed in its location throughout the Early Natufian occupations [14, 16]. Acknowledging the importance of this axis, we can suggest a reconstruction of the missing eastern part of Wall 51 to complete a 180 degrees structure open to the North. This part of the Wall 51 was not preserved probably due to the successive construction of structure 121 of Layer II. Posthole 139 (the seventh posthole that does not lie on the concentric arch) might indicate a second ring of postholes following a suggestion by Perrot [15] or, as we suggest, it could have been built at a spot where additional support was needed during the lifetime of the shelter. This posthole played a significant role in Valla’s reasoning towards his reconstruction. Yet, as a part of his suggested upper structure, this post is structurally redundant, an unnecessary structural element that was rightfully not part of reconstructing the symmetric northern part of the roof. We suggest that Posthole 139, which also differs in nature from the other postholes and lies in burial ground B, should be understood as a later addition not pertaining to the same construction episode of the other postholes. As such it was not used for calculating the center value of the series of postholes.

We have found through the spatial analysis that Wall 51, the postholes and the hearths are all arranged in a rather strict geometric relation to each other. This characteristic that supports our initial hypothesis regarding the contemporaneity of these features should not be dismissed as coincidental. We tend to accept the pre-assumption that Shelters 131 and 51 had a similar general layout. However, the extent of the Upper Floor 131, detected in squares P,Q,R,S/4-5, P/6 and N,O/6-7 as published by Valla [14], does not better support the prevailing reconstruction over the one we proposed here, which is in accordance with stratigraphic, architectural and geometric considerations (note the shaded floor in Fig 2). It seems to us that our suggested reconstruction shows a high level of parsimony both because it is based on a small number of assumptions compared to other suggestions and because most of its parts are based on existing features as published by the excavators with only a minimal use of reconstructed elements (nearly 80% of the suggested superstructure can be reconstructed without the addition of posts and perimeter wall segments). Moreover, our suggested reconstruction has an added value that might shed light on an important issue in Natufian stone construction–that is the geometric element, previously discussed by Valla and Samuelian regarding the Final Natufian architectural remains at the site [8, 9].

A tendency towards geometry was observed by Valla and Samuelian in the planning of some of the Final Natufian shelters unearthed during the last series of excavations at the site by Valla and Khalaily. They have indicated the setting of hearths and postholes on the main perpendicular axes of the shelters, suggesting that this practice was a continuation of an Early Natufian tradition. Our suggested reconstruction supports this view. Not only has it confirmed the connection between the direction of the slope and the orientation of the shelters, but also the application of a similar organizational concept, that is the role of the principal perpendicular axes. There is however a basic difference between the Early Natufian Shelter 51 and the Final Natufian structures and their conceptualization as geometric constructs. Samuelian et al. [9] and Valla et al. [8] emphasized that the geometric design of the Final Natufian constructions was only approximate and that no perfect symmetry and regularity were reached—"…the geometrization of the construction is only approximate. No perfect symmetry was reached but the tendency to follow a geometric pattern is clear." [9]; "But this tendency doesn’t reach a point where a strict regularity would be imposed on buildings. None of the examples at hand exhibit a full geometric perfection and there is a range of variability accepted in the basic scheme." [8]. On the other hand, our analysis and reconstruction of Shelter 51 reveals a geometric construction in the full sense of the term.

## Discussion

**Fig 8:**
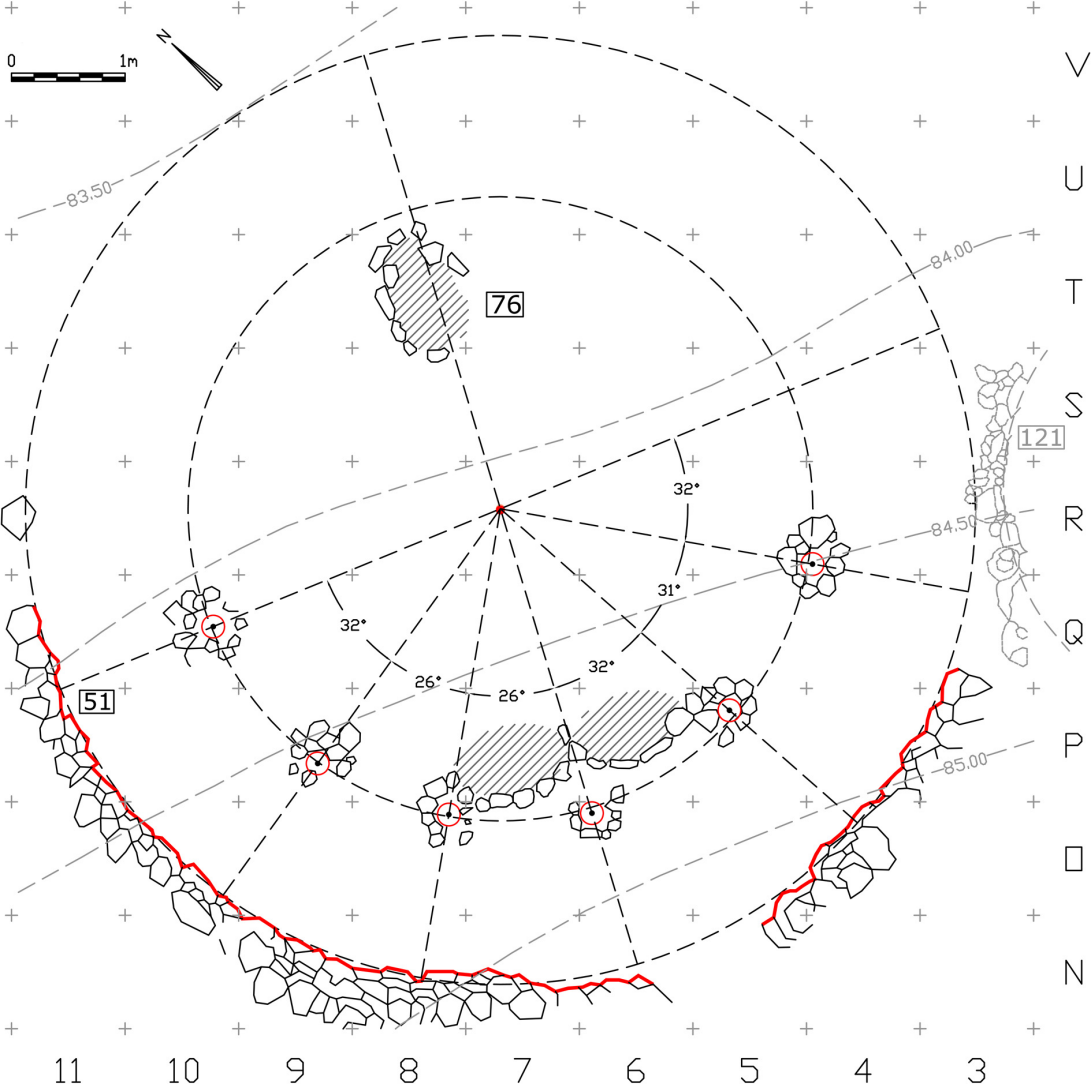
Architectural-geometrical analysis and results.

**Fig 9:**
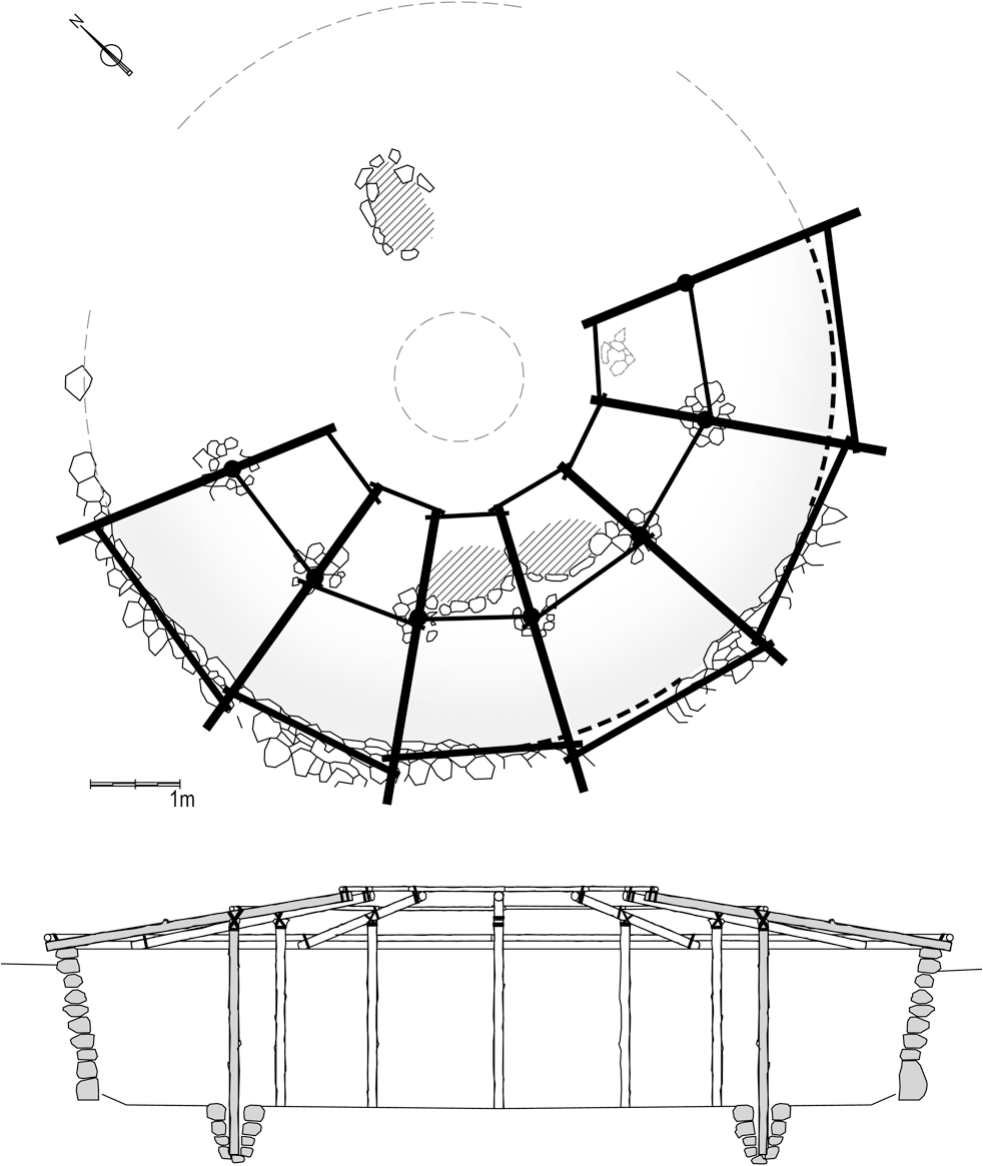
Proposed reconstruction of Shelter 51. (note the single reconstructed posthole in the northeastern part).

We Suggest that the accuracy in which the curved Wall 51 follows a circular path along a wide angel of 150 degrees (and if reconstructed the way we suggest, 180 degrees), the fairly regular rhythm of the postholes, and the exact concentricity discussed above, are all reflective of the intention to produce a symmetrical circular space, possibly by applying measuring techniques such as a compass arm (Fig 8). The Northern half of it could have been a front open space defined by stones that did not survive, accept for a few stones at the western edge, while the construction of the actual walls was limited to the southern semicircle where the walls functioned as ground retaining walls (Fig 9) (S3 Fig). We therefore conclude by suggesting that Shelter 51 had a "true" semi-circular shape, embedded into the slope and facing north. Consistent with the circular morphology and structural logic, the roof structure could have been composed of inclined radial beams supported by the perimeter wall and the posts. The elevation of the wall, reconstructed to 1.7m above floor 51 (S4 Fig), is confirmed, if we accept Perrot’s stratigraphy nearby the eastern remains of Shelter 26 indicating the sloping ground level (note the elevation notation of Wall 26 in N/11, Fig 2). The proposed reconstruction might support the interpretation of this structure as a communal meeting place (with a possible connection to the burials) rather than a dwelling [19, 20]. But not less importantly, it sheds new light on the architectural remains which now seem to reflect a precise execution of a well-defined plan, a plan that defines the relationship between different structural elements and can be called Architectural with a capital A.

**Fig 10:**
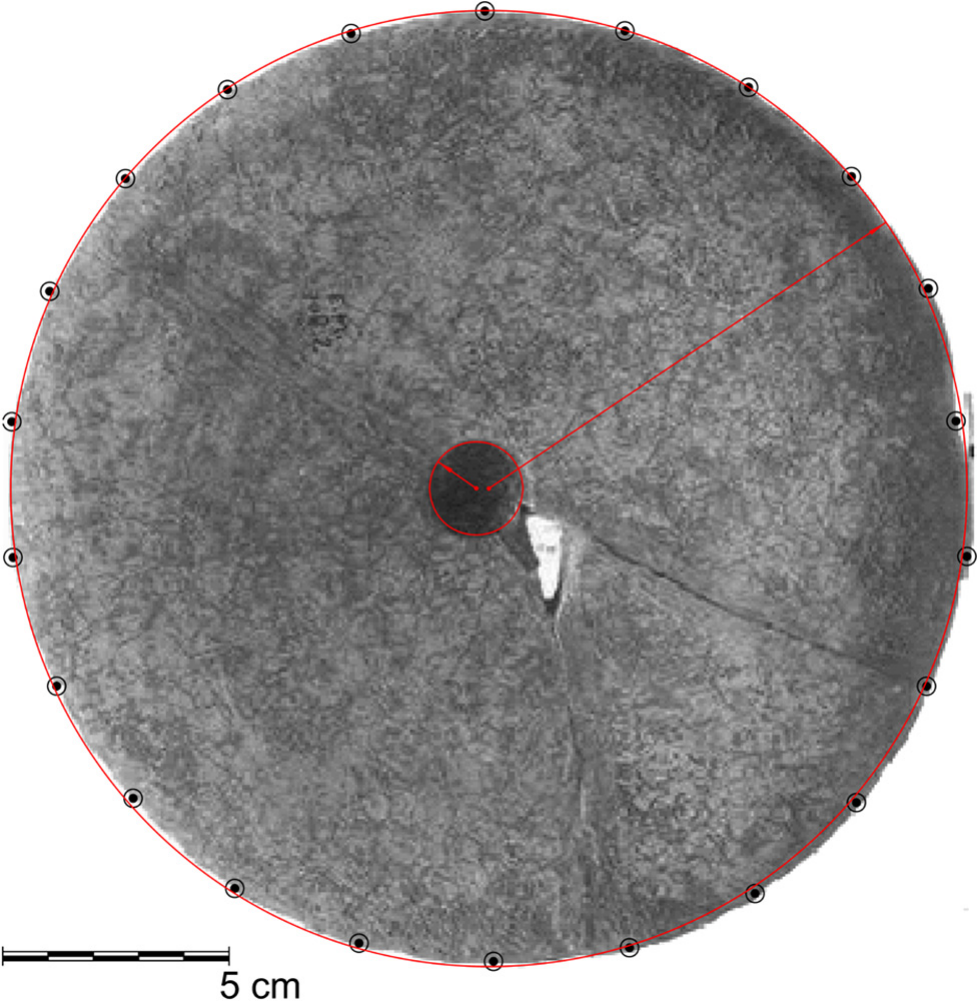
Perfect circular limestone disc. An example of a perfect circular limestone disc found at the site of Eynan (21cm diameter). Curvature analysis overlying a photograph. Collection of Israeli Antiquities Authority. Photo by the Israeli Museum, Jerusalem.

While it is not plausible to assume that amorphous shapes and irregular curves, such as the contours of the Epipaleolithic huts from Ohalo II [21], or the complex "horse-shoe" curve proposed by Valla for Shelter 131 [14], were specified in advance, it is much easier to accept a claim that the geometric shape, here suggested for the reconstruction of Shelter 51, was indeed specified and pursued (S5 Fig). Concepts and words such as semicircle and circle-center, made it possible for the people of Eynan to construct a stable and communicable mental representation of the architectural design. The Natufians were also familiar with the notion of a "perfect" and precise circle. It is evident, for example from the 62cm high mortar and stone discs retrieved from the site of Eynan [11]. These artifacts possess a strikingly high degree of symmetry (Fig 10), and reflect the intention and ability of producing objects of such properties. But when it came to space and architecture, this desire for a circular form, combined with the desire to plan in advance which is automatically followed by the desire to execute the plan with precision, would have been answered by different ways of measuring the ground.

The notion that the Natufian culture marks a fundamental change in man's relation to his environment has been recently challenged. It has been suggested that early Epipaleolithic man's relation to the brushwood huts of Ohalo II [22] or the semi subterranean huts of Kharaneh IV [23] was not very different than early Natufian's relation to stone architecture, including multiple reoccupations, ritual activities, symbolism and burials [24]. We suggest, however, that at least one important aspect of relating to the environment shows a significant change in the Early Natufian. The remains of brushwood huts undoubtedly reflect a certain level of architectural planning. Size, orientation, location of the entrance and functional organization were most probably predetermined [22]. The same argument, though, cannot be made regarding the spatial form of these huts which was clearly less planned in these cases and partially improvised as a result of construction techniques that produced structures of unique shapes. The Natufian example from Eynan reveals a whole new level of architectural design. Here, the designer addressed and integrated the different aspects of architectural planning, including spatial organization, structural system and spatial form, under a common geometric concept. This resulted in a standardization of the structural and spatial elements. Unlike the early Epipaleolithic brushwood huts, Shelter 51 was envisioned by its designer in its totality and in a different level of details. Thanks to the use of geometric concepts, a shape of a floor plan could have been defined and specified prior to its marking on the ground, and an architectural design could have been shared with others and carried out with accuracy. As worded by Marx relating to human productivity: “A spider conducts operations that resemble those of a weaver, and a bee puts to shame many an architect in the construction of her cells. But what distinguishes the worst architect from the best of bees is this, that the architect raises his structure in imagination before he erects it in reality” [25] (Ch. 7, published originally in 1867 in German).

In conclusion, we argue that the amorphous (or if one wishes, the geometrically complex) contour lines, characterizing pre-Natufian hut remains (e.g. [26], Figs 2 and 3, [24], Fig 3), seem to exclude the possibility of spatial form specifications as part of a pre-construction design process and of the use of geometry. On the other hand, the geometric regularities in which the different features of the Early Natufian Shelter 51 are arranged are convincingly explained as architectural planning rather than as a gradual accumulation of features or a self-organizing process. The Natufian level of architectural planning, an innovation made possible by the introduction of a geometric tradition, represents a turning point in human/environment relations, as the role of geometry in architectural design and its manifestation in the spatial form of the built environment were destined to become predominant.

### End Note

**Fig 11:**
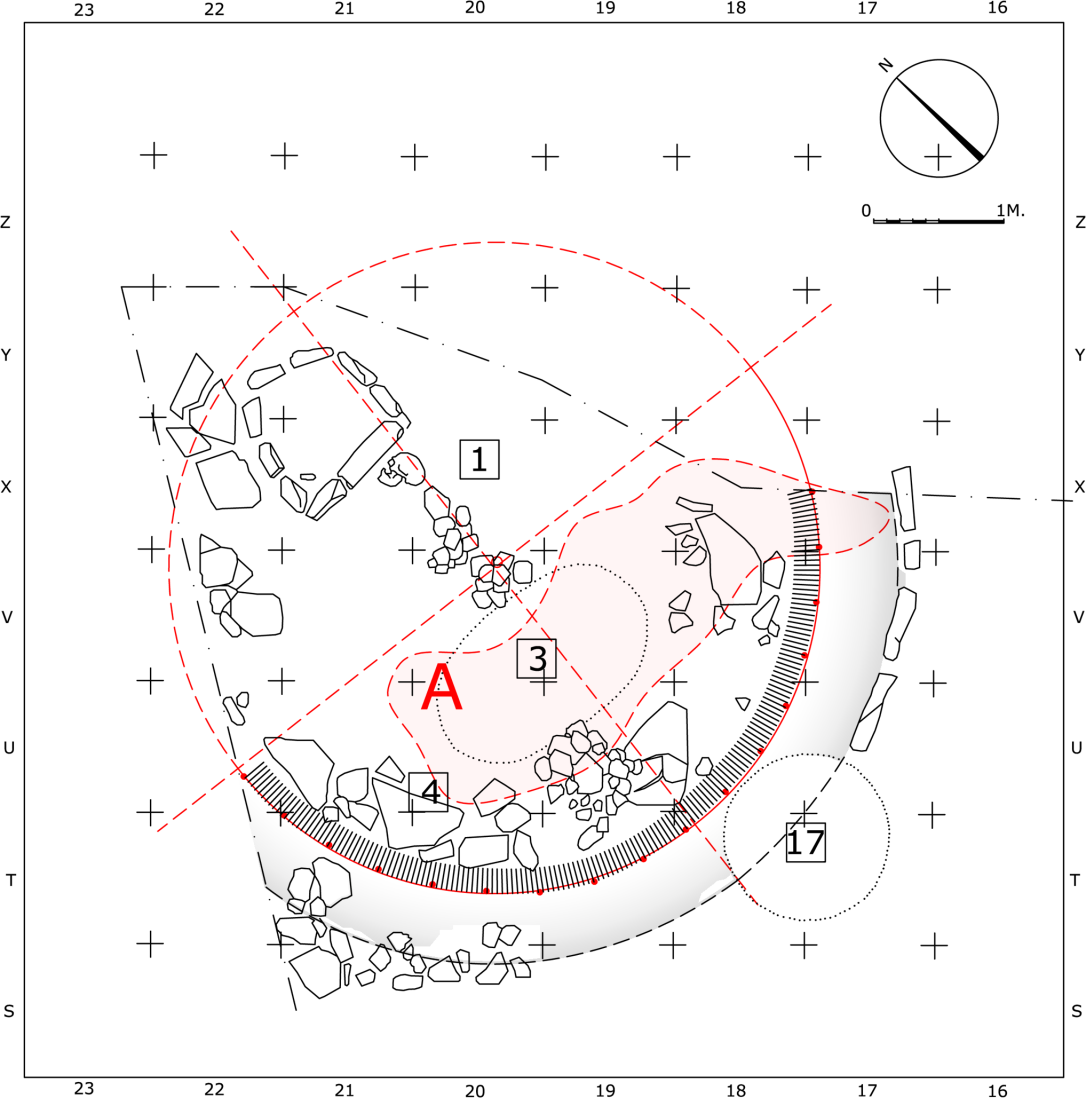
Analysis of Shelter 1. Modified from [11].

Our suggestion regarding the design process behind Shelter 51 tells an alternative story to the accepted view of “organic" "architecture without architects" story. Moreover, we suggest not only "architecture with architects" but geometric planning and geometric construction. Yet, our suggestion relates to a complex of special dimensions and possibly a special function. It thus goes without saying that our suggestion that a geometric tradition had been established in the Early Natufian, does not imply that it was necessarily applied in every structure of Natufian sites or structures built since. It may have been limited to communal or special purpose structures. Such claim has been made in the past for Shelter 131 suggesting it was a social/ritual space pertaining to the presence of burials of Cemetery B [20], and for Shelter 1 in relation to Cemetery A [13]. It is yet to be elaborated, but it seems that the design and construction of Shelter 1 involved similar geometric principles (Fig 11).

It has been suggested that architectural resemblance between the public structure of Eynan and several post-Natufian, Pre-Pottery Neolithic A communal structures of the northern Levant may indicate a cultural continuation from the Natufian (e.g., [27]). In fact, some of these structures reflect a rigorous geometric design as well. Such is the case for Jerf El-Ahmar, structure EA 53 where the posthole arrangement was described by Stordeur as forming a perfect equilateral hexagon that fits harmoniously into the circle of the building ([28] and refs therein). This structure clearly reflects a very detailed planning. The very presence of such communal buildings that possibly reflect the state of the art of architectural design of their time, suggests that the story of architectural design and construction technology should devote an important chapter to these exemplary projects.

## Supporting Information

S1 Fig Curves superimposed on a drawing by Perrot [18]. (TIF)

S2 Fig Circle fitting algorithm. In this algorithm, a grid is superimposed over the analyzed area. For each point of the grid, the standard deviation of the distances to the given points and the mean distance are calculated. The algorithm searches for the center point in which the standard distance deviation is minimal.
(TIF)

S3 Fig Reconstruction of Shelter 51. Perspective view.
(TIF)

S4 Fig Generic section. Showing the reconstructed and actual elevation found in the field of wall 51 (at its highest point, note different shades of the stones), and of the estimated sloping ground level.
(TIF)

S5 Fig Prevailing and suggested reconstructions. (a) The Valla reconstruction of Shelter 131 (modified from [14]) overlying the Early Natufian architectural remains and burials (note the three reconstructed postholes); (b) The suggested reconstruction of Shelter 51 overlying the Early Natufian architectural remains and burials (note the single reconstructed posthole in the northeastern part).
(TIF)
