# Supplementary figures and images for: A New Look at Shelter 131/51 in the Natufian Site of Eynan (Ain-Mallaha), Israel
Source: PLoS One. 2015 Jul 8;10(7):e0130121. doi: 10.1371/journal.pone.0130121 (PMC4496039; doi:10.1371/journal.pone.0130121)

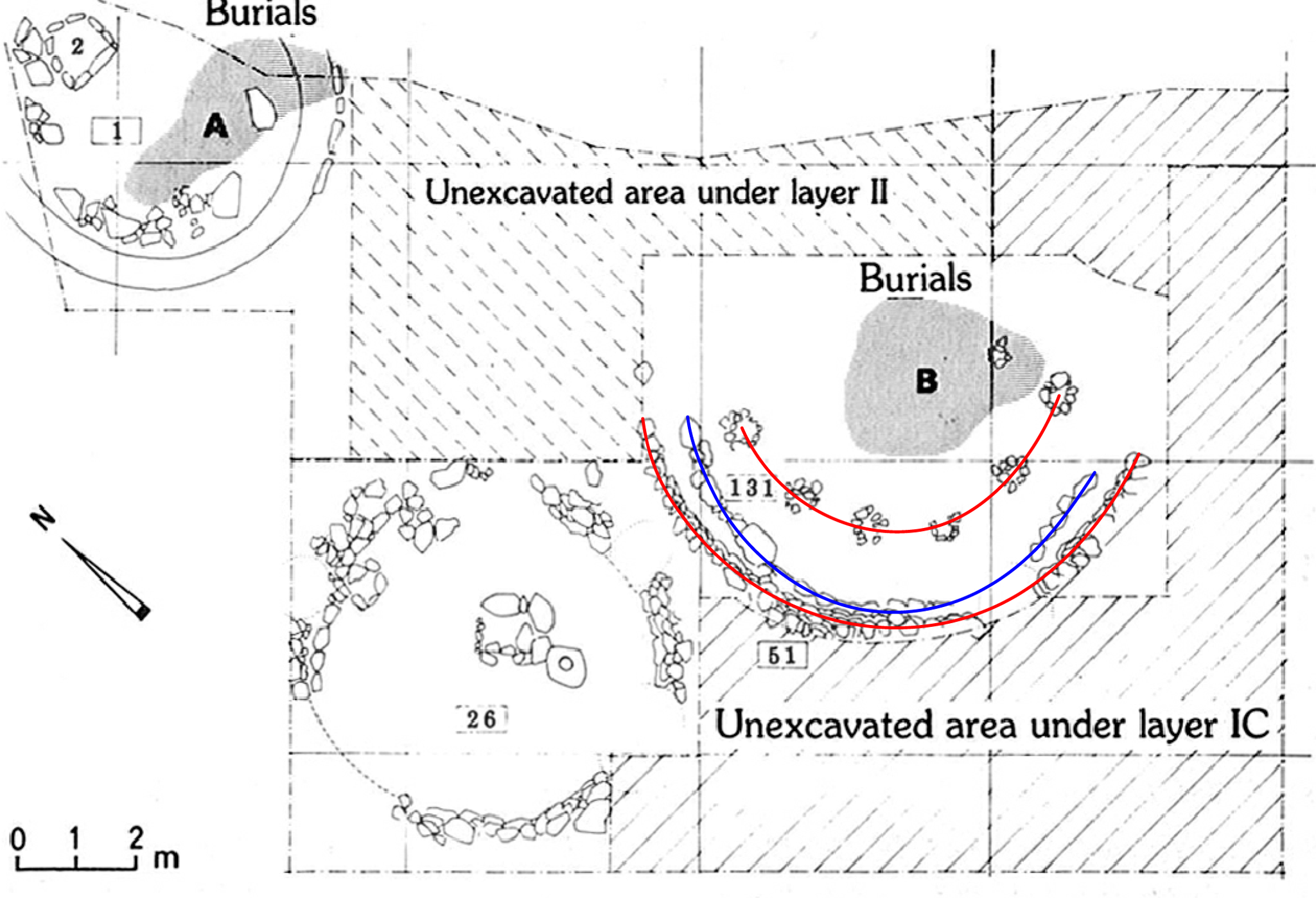

Supplement: S1 Fig — (TIF) [file pone.0130121.s001.tif]

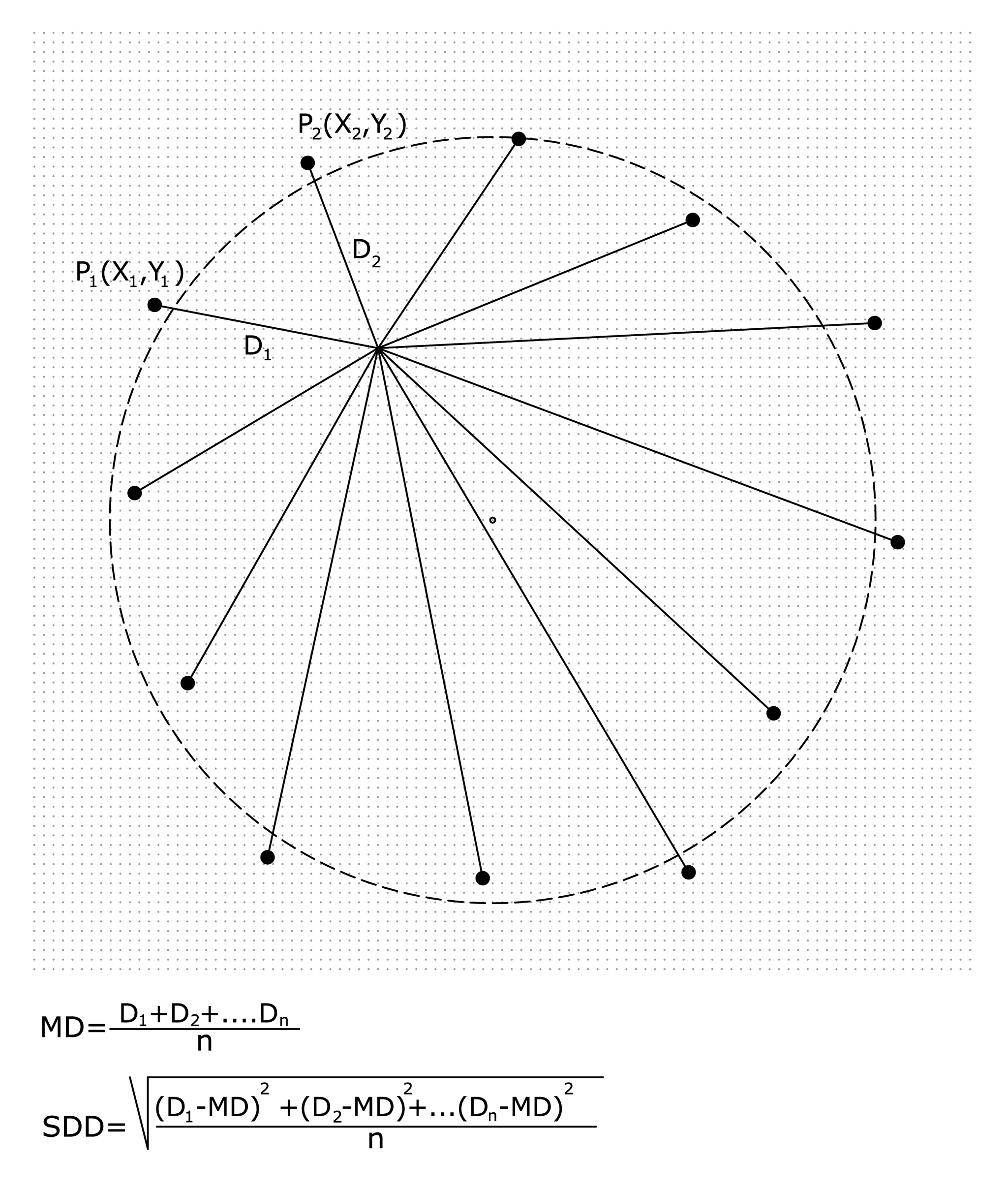

Supplement: S2 Fig — In this algorithm, a grid is superimposed over the analyzed area. For each point of the grid, the standard deviation of the distances to the given points and the mean distance are calculated. The algorithm searches for the center point in which the standard distance deviation is minimal. (TIF) [file pone.0130121.s002.tif]

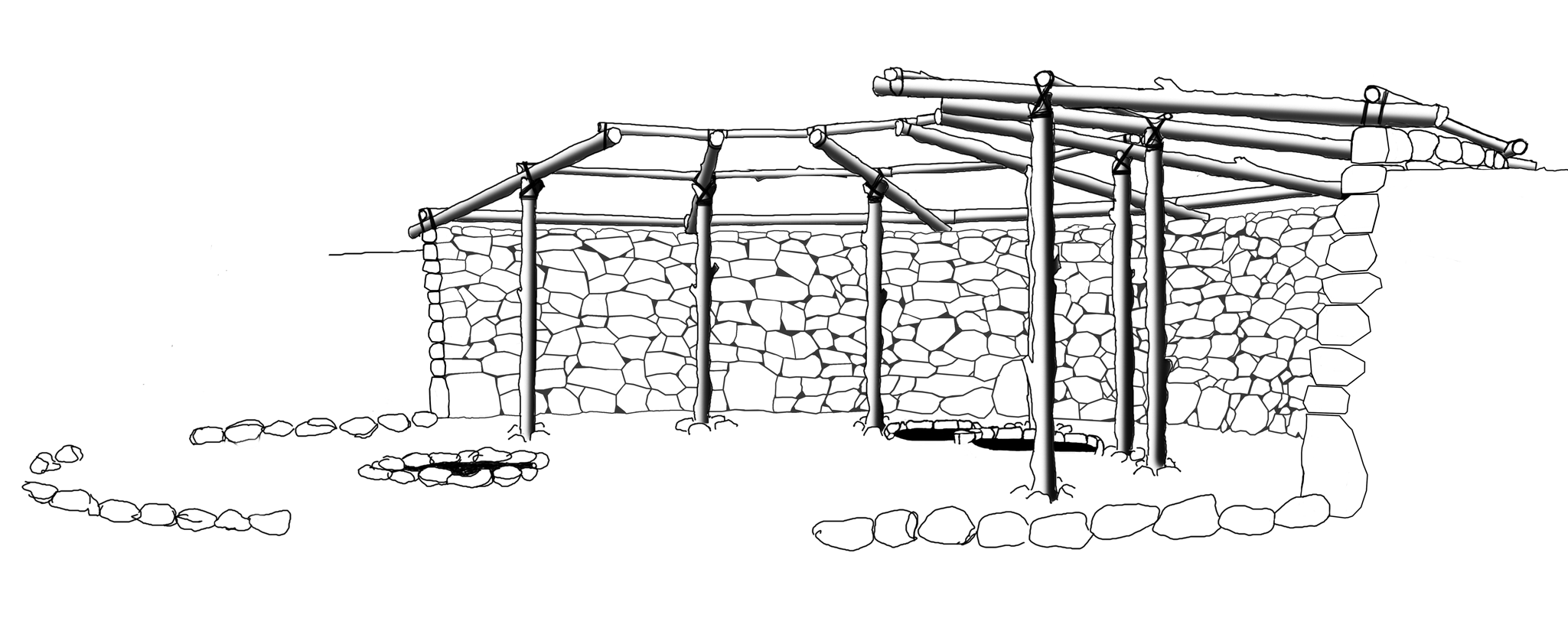

Supplement: S3 Fig — Perspective view. (TIF) [file pone.0130121.s003.tif]

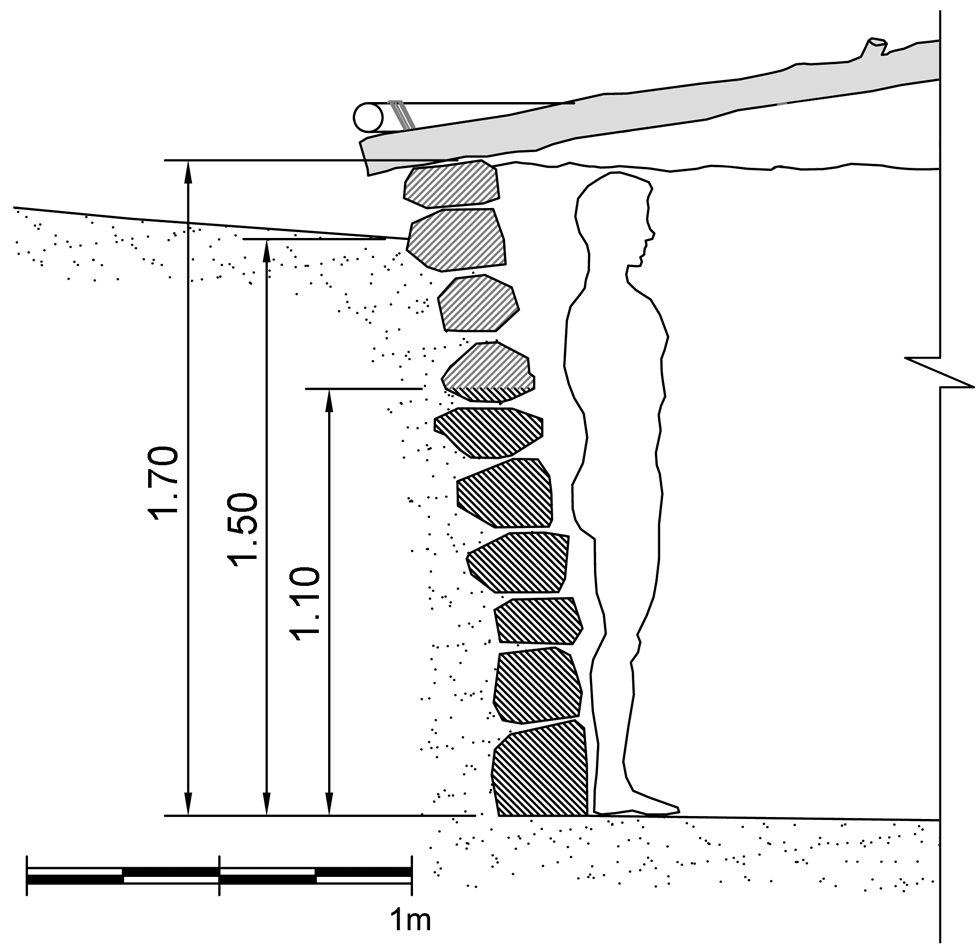

Supplement: S4 Fig — Showing the reconstructed and actual elevation found in the field of wall 51 (at its highest point, note different shades of the stones), and of the estimated sloping ground level. (TIF) [file pone.0130121.s004.tif]

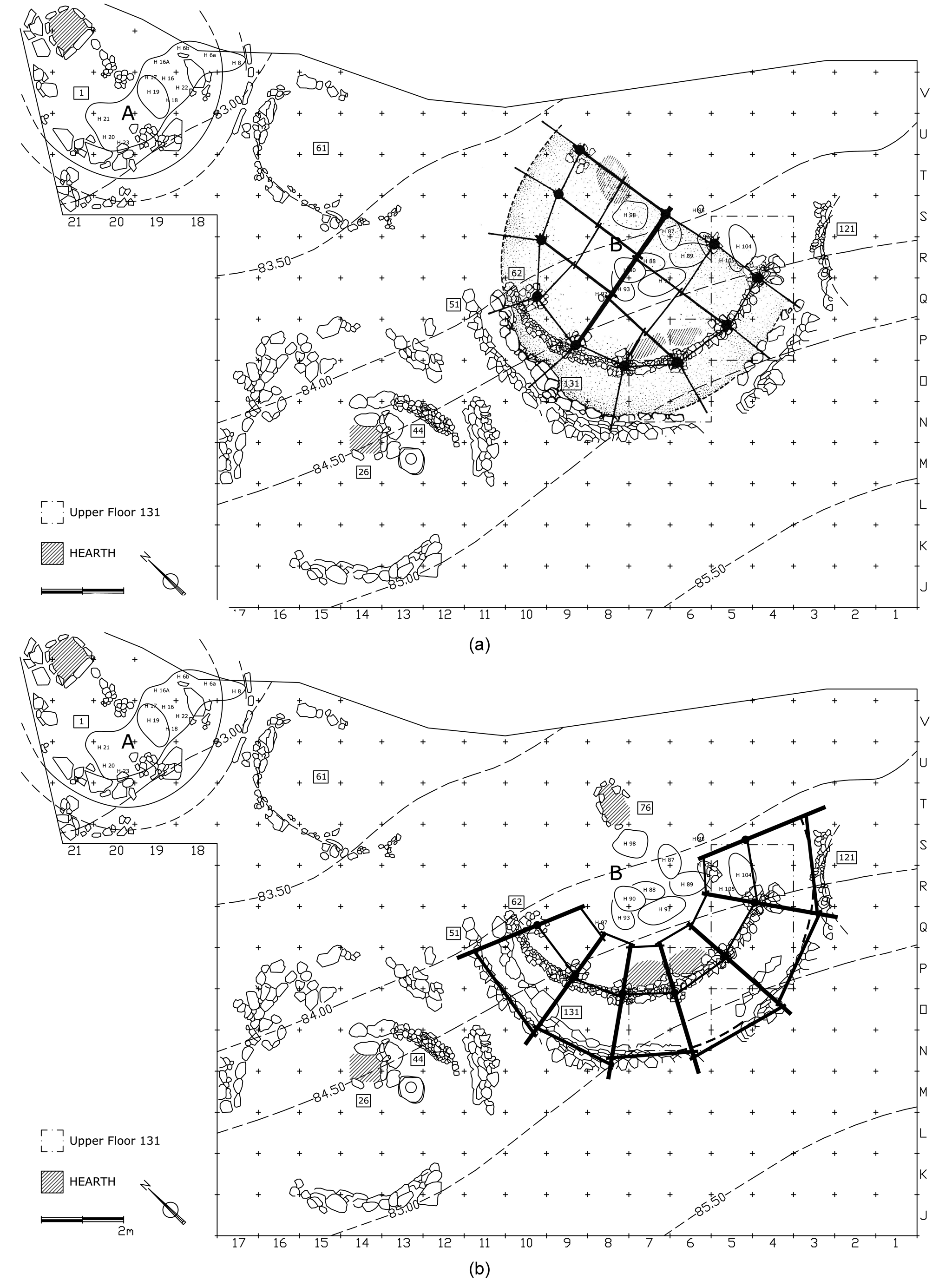

Supplement: S5 Fig — (a) The Valla reconstruction of Shelter 131 (modified from [14]) overlying the Early Natufian architectural remains and burials (note the three reconstructed postholes); (b) The suggested reconstruction of Shelter 51 overlying the Early Natufian architectural remains and burials (note the single reconstructed posthole in the northeastern part). (TIF) [file pone.0130121.s005.tif]
